# Tsetse Control and the Elimination of Gambian Sleeping Sickness

**DOI:** 10.1371/journal.pntd.0004437

**Published:** 2016-04-29

**Authors:** Mike Lehane, Idriss Alfaroukh, Bruno Bucheton, Mamadou Camara, Angi Harris, Dramane Kaba, Crispin Lumbala, Mallaye Peka, Jean-Baptiste Rayaisse, Charles Waiswa, Philippe Solano, Steve Torr

**Affiliations:** 1 Liverpool School of Tropical Medicine, Liverpool, Merseyside, United Kingdom; 2 IRED, B. P. 433, N'Djaména, Chad; 3 IRD, UMR 177 IRD-CIRAD INTERTRYP, PNLTHA-Ministère de la Santé, Conakry, Republique de Guinee; 4 PNLTHA, Ministère de la Santé, Conakry, Republique de Guinee; 5 Bill and Melinda Gates Foundation, Seattle, Washington, United States of America; 6 Institut Pierre Richet / Institut National de Santé Publique, BP V 47 Abidjan, Côte d’Ivoire; 7 National Human African Trypanosomiasis Control Program, Kinshasa, Democratic Republic of the Congo; 8 PNLTHA, Ministry of Health, N'Djaména, Chad; 9 CIRDES URBIO, 01 BP 454, Bobo-Dioulasso, Burkina Faso; 10 Department of Biomolecular and Biolaboratory Sciences, School of Biosecurity, Makerere University, Kampala, Uganda; 11 IRD, UMR 177 IRD-CIRAD INTERTRYP, CIRDES 01 BP 454, Bobo-Dioulasso, Burkina Faso; Lancaster University, UNITED KINGDOM

Sleeping sickness, or Human African Trypanosomiasis (HAT), is caused by two distinct parasites. In East and Southern Africa, *Trypanosoma brucei rhodesiense* causes the Rhodesian form of the disease (about 2% of all reported cases [1]). In Central and West Africa, *T*. *b*. *gambiense* causes the Gambian form of the disease (G-HAT—about 98% of all reported cases [1]). The disease normally affects remote rural communities. The people most at risk are those working outdoors for long periods, as they are most exposed to the bite of the tsetse fly (*Glossina* spp.: Diptera), which transmits the parasites. The comparable diseases which occur in livestock, collectively termed African Animal Trypanosomiasis (AAT), are a significant brake on African development [2]. Among the 31 tsetse species, the most important vectors of G-HAT are *Glossina fuscipes* and *Glossina palpalis*, which are riverine tsetse species (Palpalis group).

Since the start of the 20th century, HAT has occurred in three huge epidemics. The most recent was in the 1990s when the annual cases officially reported to WHO peaked at 37,385 in 1998. It is widely acknowledged this severely underestimated actual numbers infected, which may have been as high as 450,000 in 1999 [3]. Untreated disease is normally fatal, so undoubtedly, many people infected in these epidemics died as a result. Although treatments for the disease have improved [4], they are still complex and difficult to administer particularly in the resource-poor settings where the disease thrives. There is no vaccine or chemoprophylaxis to prevent HAT and little prospect of either being developed in the near future. Vector control therefore remains the only means of protecting people from infection.

Rhodesian HAT (R-HAT) is a zoonosis. As a consequence, vector control plays a key part in its control, and medical interventions are only used for humanitarian purposes. In contrast, G-HAT is generally considered to be an anthroponosis, and control has relied heavily on active and/or passive case detection and treatment programmes [5]. However, modelling [6], historical investigations [7], and practical interventions [8,9] have clearly demonstrated the role that vector control can play in control of G-HAT, but it was considered too expensive and difficult to deploy in the resource poor settings of HAT foci. In consequence, a study was started in 2006 to try to find a simpler and cheaper alternative for vector control suitable for G-HAT foci.

The original hypothesis was that modifying insecticide-treated targets was the most likely means of producing a more cost-efficient vector control method for use in G-HAT foci. Two separate approaches were tried—to develop odours for use with targets or to change the visual characteristics of the target. The crucial finding was that a tiny target consisting of a small square of blue cloth flanked by a similar sized piece of black netting (Fig 1) was highly effective and would be about ten times more cost-effective than traps or large targets in control campaigns for the Palpalis (riverine) group tsetse flies responsible for the transmission of the vast majority of HAT [10–12]. This is in very strong contrast to Morsitans (savanna) group tsetse flies, which require much larger targets (1–2 m^2^). Importantly, it was found that all of the major G-HAT vectors responded well to tiny targets [13]. In addition, vegetation growth around tiny targets is a much smaller problem [14] than is the case for the large targets used against Morsitans group flies. In contrast to Morsitans group flies, odours seem to play only a minor role in the attraction of Palpalis group flies [15,16]. A modelling approach suggests that habitat geometry is the reason why Palpalis group flies are more dependent on sight than odour [17]. The general expectation is that relatively immobile insects in restricted habitats are more dependent on a thorough, vision-based search of their environment and that they are more wide-ranging in their diet.

**Fig 1:**
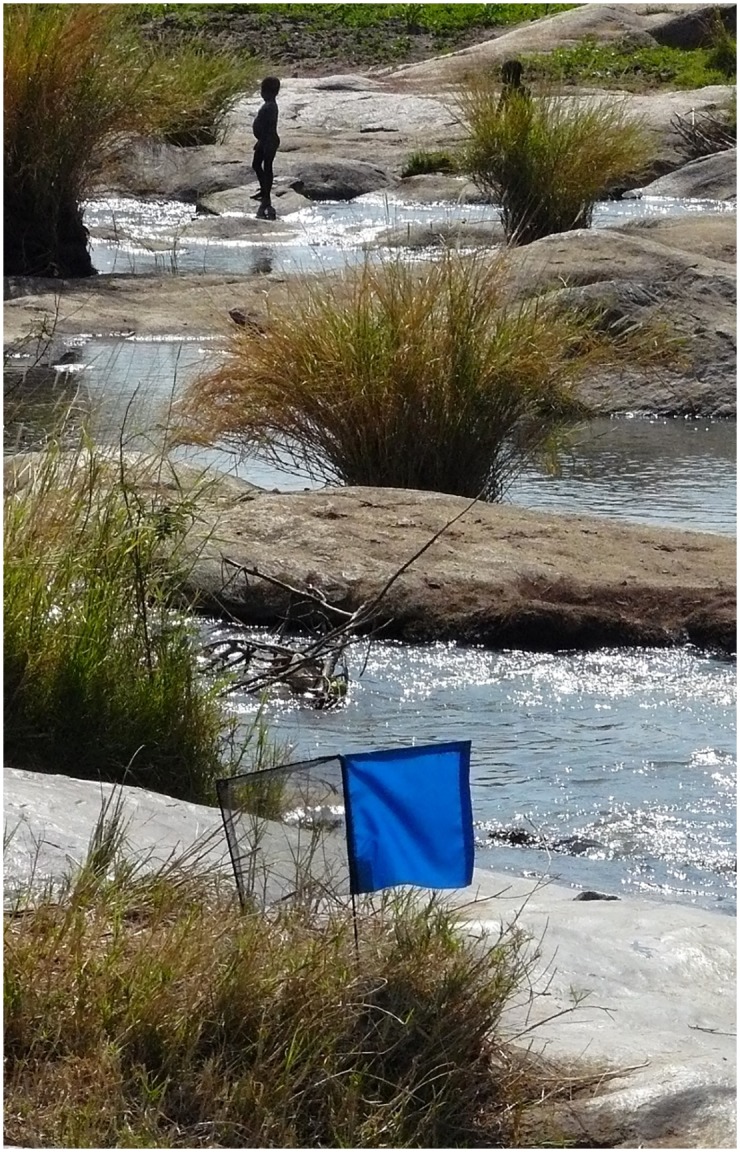
A tiny target in a typical setting in Uganda.

Inevitably, the targets are gradually degraded by challenges in the environment, and the worst problems are floods, fallen targets, and the 6-month effective life of the insecticide in the tiny target [18]. As a consequence, current practice has been to deploy tiny targets once or twice per year, and the method has been successful in practice [9,18].

The aim in HAT foci is not to eradicate tsetse (although eradication should be embraced if feasible), but to stop transmission by reducing tsetse—human contact, and modelling suggests that this does not require complete removal of tsetse flies [6]. In addition, the reported time course of disease in humans is typically 3–4 years so that a fixed period of interrupted transmission may be sufficient to eliminate HAT in a focus. This approach is basically similar to the successful World Bank-funded OCP programme, which has led to the elimination of onchocerciasis as a public health problem in West Africa [19]. The approach had also been applied successfully in HAT foci of Ivory Coast in the 1980s and 1990s by Laveissière and colleagues [8], although at that time the control techniques used were not considered to be sustainable and cost effective.

Consequently, to test the utility of tiny targets, studies were started in G-HAT foci (typically 500–3,000 km^2^). To re-emphasise, the goal is to reduce tsetse numbers below a threshold for transmission for a defined period to either eliminate or reduce transmission in a HAT focus, thereby giving screen-and-treat programmes a far greater chance of success [20]. For example, a previously published model [6] has been used along with figures from West Nile, Uganda to calculate the impact of various levels of vector control on transmission in that region (Fig 2) [18]. In practice, the level of control actually achieved in that region was >90%, which exceeds the levels required to interrupt transmission (Fig 2) [18]. How long control must continue is a researchable question but, given the time course of the disease in humans, it is likely to be several years. Presumably, it is also dependent on the distribution of the parasite in the human population and/or the existence of reservoir hosts. Current discussions have been focusing on 4–5 years of control.

**Fig 2:**
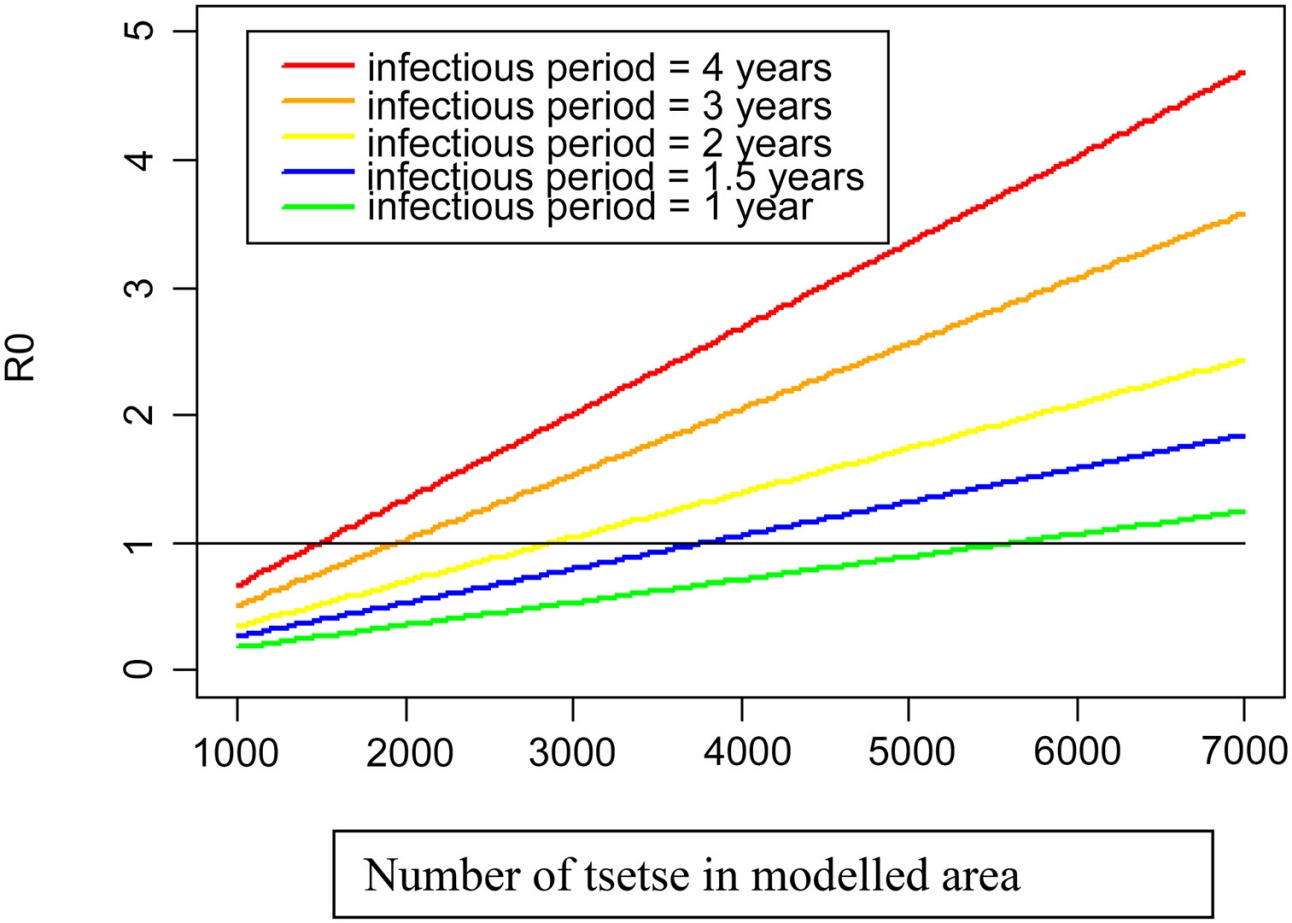
To obtain an estimate of the level of tsetse control required to stop transmission, a published model was rearranged [6]. The chart shows the relationship between HAT transmission (R0, *y*-axis) and numbers of tsetse (*x*-axis), when the average infectious period in humans is 1–4 years. The other parameters used in the model are for the West Nile region of Uganda [18]. The average infectious period is often accepted as 3 years, and so it can be seen that a reduction in tsetse numbers of approximately 72% is required to drive R0 < 1 in these settings.

## Effectiveness of Tsetse Control Using Tiny Targets

The two largest tests of tiny targets in G-HAT foci have been carried out in Uganda (*G*. *fuscipes fuscipes*) and Guinea (*G*. *palpalis gambiensis*). In Northern Uganda, a trial was performed over 500 km^2^ covering two HAT foci. Nearly 3,000 targets were deployed every six months giving an overall target density of 5.7/km^2^. In 12 months, tsetse populations declined by more than 90% [18]. In Guinea, the studies were used to determine the efficacy of adding vector control to screen-and-treat in the Boffa HAT focus. The focus was divided into two parts [9], and active screen-and-treat was carried out in the western part, whereas in the eastern part active screen-and-treat was combined with vector control. In the area with vector control, there was an overall 80% decrease in tsetse density resulting in a significant decrease of human tsetse contacts [9]. This was reflected in a decrease of disease incidence (from 0.3% to 0.1%; *p* < 0.01) with almost no new infections occurring (<0.1% in one year). In contrast, in the area with medical interventions but no vector control, incidence was ten times higher (>1%, *p* < 0.0001), and disease prevalence increased slightly (from 0.5 to 0.7%, *p* = 0.34) [9].

As we can see, the method works—but is it affordable? The costs of the Uganda operation were studied in depth [21]. The overall cost of tiny targets for control in this setting is US$85 per km^2^ per year, and this is over five times cheaper than trap-based methods used previously in Uganda (Table 1) [22]. Partly, this is due to the comparatively low cost of the tiny target itself but also to the fact they are much easier to deploy. The area being controlled has a population density of approximately 500 per km^2^ according to the Ugandan National Census. If we adopt a simple arithmetic approach, the cost for a single round of active case detection (excluding treatment), covering 80% of the population, is US$433,333 (based on WHO figures). The economic analysis of the vector control programme has been performed [21] and shows that it costs US$42,700 per year. We are not arguing for the replacement of active case detection by vector control. Instead, we wish to see this method of vector control added to case detection and treatment programmes, and the argument for doing so is strong [18]. Costs are also being calculated for vector control operations in Chad and Ivory Coast. These interventions are also likely to have the added benefit of an impact on AAT in the area, and in some cases (e.g., Mandoul, Chad) the effect could be considerable [23].

**Table 1:** Comparative costs of tsetse control operations.

1	2	3	4	5
Traps/Targets	ITC	SAT	SIT plus	Tiny Targets
US$482	US$220	US$552	US$993-US$1,365	US$85

The calculations assumed the use of four insecticide-treated traps or targets per km^2^. The costs of using traditional tsetse control methods are shown (columns 1–4). These have been calculated for a hypothetical operation in Southeast Uganda [22], and the summary figures are given here. The figures were calculated for creating a tsetse-free zone against isolated populations of tsetse flies (i.e., where reinvasion is not an issue) [22]. The calculations assumed the use of four insecticide-treated traps or targets per km^2^; ITC: restricted application of insecticide on 5 cattle per km^2^; SAT: aerial spraying of insecticide based on the Okavango programme; SIT: the recommendation for the sterile insect technique that it is used after suppression is achieved by one of the previous three methods—addition of SIT would cost an additional US$758 per km^2^. In addition, the costs for use of tiny targets have been separately calculated (column 5; note, these are not for a tsetse free zone) [21].

### The management of control programmes

For most control operations, the pressure is to deliver programmes rapidly and at scale. In our opinion, this will be best achieved in a top-down approach. To date, a range of methods has been used to suit the government arrangements, which are in place. In Uganda, control operations have been closely integrated with the central government institution, COCTU (Coordinating Office for the Control of Trypanosomiasis in Uganda), which ranges across the ministries of Health and Agriculture. Uganda already has a network of District Entomologists (DE) throughout the country. Using COCTU as the central organising influence, with some outside technical assistance, and using the DEs to organise target distribution locally, the system is working well [18]. In an ongoing trial in Chad, control operations have been integrated with the livestock research institution for development (IRED) with coordination from the Ministry of Health (PNLTHA), who have assembled field teams to deploy targets. With some outside technical support, tsetse control in the Mandoul focus is again working very effectively, and the results will be reported in 2016. In Guinea, control operations have been integrated with the PNLTHA from the Ministry of Health. With some outside technical support this central structure works with the peripheral health structures, such as the Direction Préfectorale de la Santé in the focus of Boffa along with additional agents in each village. We can see that other governments might wish to use nongovernmental organizations (NGOs) to deliver effective vector control as is common with bednet distribution and with the delivery of indoor residual spraying campaigns. In summary, like bednets or mass drug administration, the technology is seductively simple, but organising deployment is not—the key factor in success is managing deployment in a cost and operationally-effective manner. With cost effectiveness in mind, it may be important to consider here how vector control might be best integrated with screening and treatment.

### Outlook

The epidemic in the 1990s focussed attention once more on HAT. There has been great progress, and the reported annual global incidence is now < 5,000. This has increased the donor focus on the disease, and considerable funding has been provided to try to improve three major tools for use in control—new drugs, diagnostics, and more cost-effective vector control techniques. Thankfully, all three are showing great promise, and the international community wants to move towards elimination of G-HAT [24]. Modelling clearly suggests we will need vector control as well as screen-and-treat to achieve this in a reasonable time frame [25,26]. We believe that the critical decisions on whether a large international effort against G-HAT will be mobilised or not are currently in the balance, and it is our opinion that vector control will need to be a central plank in any programme in addition to medical activities.

### Dedication

To the memory of Ali Bachr Alkatib, who lost his life in the control operations in Chad.
